# Quadrigeminal plate lipoma presenting with Psychosis: A case report with review of literature

**Published:** 2015-09

**Authors:** Sourav Das, Manju Saini, Mohan Dhyani, Ravi Gupta

**Affiliations:** 1. Departments of Psychiatry and Radiology Himalayan Institute of Medical Sciences, Swami Ram Nagar, Jolly Grant, Dehardun-248016, India

A young patient who presented with headache followed by positive and negative symptoms of psychosis and mutism was sent for the MRI of brain. MRI revealed a lipoma in the quardrigeminal area. We hypothesized that the neuro-vascular encasement of structures located at the upper dorsal midbrain by the lipoma caused the symptoms. A review of the current literature of quadrigeminal lipoma cases with presenting symptoms is provided. Lipoma in quardrigeminal area could give rise to symptoms of psychosis.

Intracranial lipomas are extremely rare benign tumors, accounting for <0.1 % of all primary brain neoplasms (1). Most commonly, they occur in the trigonal choroid plexus on cerebral convexities in the pericallosal area, suprasellar/interpeduncular cistern, cerebellopontine angle cistern, sylvian cistern and in quadrigeminal cistern regions (2, 3). Most of them are asymptomatic, usually discovered as incidental findings during autopsy or brain imaging (4). Rarely, they may be symptomatic and found to be associated with seizures or headache (5). Here, we present a case of a teenage female presenting with symptoms of psychosis whose MRI scanning showed a quadrigeminal cistern lipoma. We believe that this is the first reported case ever of such a lesion presenting with psychosis.

## Case report

The case was a 17- year- old unmarried female patient studying in 9th standard from a low socio-economic background without any significant past or family history presented with symptoms of irrelevant talks, muttering, smiling to self without any apparent reason, disorganised behaviour and poor self-care for the last one month.

Her symptoms started gradually three months ago when she complained of persistent, constricting type of diffuse headache of mild to moderate severity, which increased when she was reading. Two weeks later, she woke up one night with a sudden cry and shouting, expressing that she felt pain all over her body as if something was pressing on her. She became restless, fearful and wandered aimlessly in the house for hours. However, her consciousness was never altered and she never became unresponsive even for a brief period of time.

Over the next few days, her restlessness continued to increase, and she became impulsive and touched and pulled others coming near her without any apparent reason. Her sleep was decreased and she showed odd behaviours like nodding her head, shaking the hands and head as well as making odd gestures. Smiling to self and drooling of saliva were also noticed, and her self-care decreased. She also micturated and defecated in her clothes and did not seem disturbed about it. There was a general loss of shamefulness and on multiple occasions she took out her clothes in public or wandered around inadequately dressed. Gradually, her talking decreased to the point of being silent. 

There was some improvement in her symptoms over the next two- three weeks spontaneously without any treatment. She started talking, although mostly irrelevantly, and was not found wandering with inadequate clothes though her clothing remained mostly dirty and dishevelled. However, her self-care continued to remain poor, she smiled to herself and muttered on occasions. She also micturated or defecated outside her house though ablution was limited and unsatisfactory. She was also noted to be sitting or standing in the same position for long durations and did not respond adequately when enquired about the same. 

There was no history to suggest seizures, delirium, drug intoxication, delusions, bipolar disorder, dissociative disorders, sleep disorders and any medical or surgical disorders. 

Mental status examination revealed clear consciousness. Her attention could be aroused easily but it was ill-sustained, and there was mild impairment in orientation. The patient was comprehending communication, as was evident by her following instructions like looking at her mother, picking up the pen etc. However, her judgement was impaired. 

**Table1:** Clinical presentation of quadrigeminal cistern lipomas

**Year**	**Author(s)**	**n**	**Age (in Years) /Sex**	**Country**	**Symptoms**
2013	Jha et al (12)	1	3	India	single episode of generalized tonic-clonic seizures
2013	Majumdar et al (13)	1	10M	India	headache since 2 years of age along with recurrent vomiting and drooping of left eyelid during the attack
2012	Khoshnevisan et al (14)	1	20M	Iran	Headache
2012	Panil Kumar et al (15)	1	32M	India	Headache and Seizures
2009	Ogbole et al (1)	1	70F	Nigeria	Headache
2008	Senoglu & Altun (7)	1	37F	Turkey	Headache
2005	Yilmazlar et al (11)	1	37F	Turkey	raised intracranial pressure
2005	Fandiño et al (4)	1	47	Spain	headache dizziness and quadrantanopsia
2002	Kiymaz & Cirak (16)	1	2F	Turkey	encephalocraniocutaneous lipomatosis
1998	Ono et al (3)	1	7M	Japan	complex partial seizures
1998	Sala et al (17)	1	4M	Italy	Epilepsy and behavioural change
1995	Nikaido et al (8)	1	65M	Japan	left abducens nerve paresis
1993	Uchino et al (18)	6		Japan	mildly dilated ventricular system in one, rest asymptomatic
1991	Howng & Chang (19)	1		China	headache; especially over the occipital area; and, blurring of vision
1989	Uchino et al (20)	1		Japan	Asymptomatic
1987	Summers et al (21)	1	10F	USA(Minneapolis)	Congenital ocular motor apraxia
1987	Maiuri et al (22)	1	62M	Italy	Intracranial hypertension
1986	Friedman et al (23)	1	63M	USA(Maryland)	Headache, blurred vision, behavioural change
1985	Ambrosetto et al (24)	2		Italy	impairment of vertical gaze in one
1983	Hayashi et al (25)	2	infants	Japan	obstructive hydrocephalus in one, agenesis of corpus callosum in other

**Fig 1:**
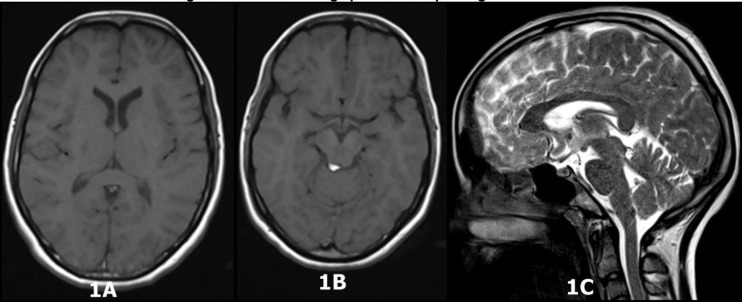
MRI Brain showing lipoma in the quardrigeminal area

1A. T1 image showing normal sized lateral ventricles

1B. T1 image showing lipoma in quardrigeminal area

1C. T2 image showing lipoma in quardrigeminal area

Her hygiene was poor with dishevelled clothes, unkempt hair, restlessness, distractibility and poor maintenance of eye contact. Her speech was hesitant and mostly irrelevant with loosening of associations, occasionally incoherent and was barely audible. Her affect was inappropriate and labile. Physical examination revealed mildly brisk deep tendon reflexes (bilateral biceps, supinator, knee and ankle jerks were Grade 3+) and equivocal bilateral planter response. Further examination was not possible.

The patient was seen by the consultant psychiatrist of the day and was diagnosed with acute and transient psychotic disorder as per International Statistical Classification of Diseases and Related Health Problems (ICD)- 10th Revision criteria (6). Her routine blood investigations and brain MRI (without contrast) were ordered. All the routine blood investigations were within the normal limits. MRI scan of the brain, however, showed a lesion (0.5cm x 0.42cm x 0.45cm) in the quadrigeminal cistern area in the posterior aspect of the right inferior colliculus of the midbrain and right superior medullary vellum and anterior to the lingula of the right cerebellar hemisphere. The lesion was hyperintense in both T1 and T2 sequences which was diagnosed as lipoma since these signal intensities were consistent with fat (7). However, fat suppression sequence in MRI could not be performed as it is not done routinely for all cases in our centre due to the very high workload. There was no evidence of any mass effect or obstructive changes in the brain parenchyma. Neurosurgical referral was sought, where conservative management was recommended. The patient was prescribed Tab. Olanzapine 5mg per day (increased to 10 mg per day after 5 days) and 1 mg of tab. Lorazepam, to which she responded within one month, and is currently on the same medication. 

## Discussion

The patient was diagnosed as a case of psychosis due to the presence of disorganized behaviour, formal thought disorder, persecutory ideas/delusions and negative symptoms in the form of abulia and poverty of speech (6). Moreover, keeping with the nature of acute and transient psychosis, it also presented with a polymorphic course. Delirium and focal seizure were ruled out by absence of alteration in consciousness, presence of adequate responsiveness and prolonged symptoms. However, some notable findings of the case were headache at the onset, slowly developing and variable mutism, a sensation described by the patient as “as if something is pressing” and soiling of clothes with urine and stool.

Headache preceding the onset of psychosis along with soiling of clothes necessitated the brain imaging in this case. The incontinence could be the urge or overflow incontinence, or it could be a part of disorganized behavior of psychosis. The “as if something is pressing” sensation in the absence of any stimulus is likely to be tactile hallucination. Explanation for both of these symptoms could not be clarified beyond doubt due to the difficulty in communicating with the patient.

The imaging findings confirmed lipoma of the quadrigeminal plate. Earlier studies have reported that further histo-pathological confirmation is not necessary to diagnose lipoma; hence, they were not sought (7). There are other differential diagnosis for lipoma in this region which include arachnoid and tectal plate cyst, tectal masses, supracerebellar abscess, dermoid and epidermoid cysts, ruptured P4 segment aneurysm of the posterior cerebral artery and also pineal region mass (8,1).

Intracranial lipomas are extremely rare developmental tumors arising from abnormal persistence and development of primitive meninges (9). Lipoma in the quadrigeminal region includes that in the quadrigeminal cistern, the quadrigeminal plate, the ambient cistern, the superior vermis, or the superior medullary velum (1, 10). In about one fifth of the cases, these lipomas can cause significant mass effects (3), with neurologic deficits, obstructive hydrocephalus, or raised intracranial pressure (11). Usually patients present with headache, dizziness, psychomotor retardation, generalized or complex partial seizures, visual disturbances or may be asymptomatic (8). A review of literature of reported cases of quadrigeminal lipoma with presenting symptoms is given in Table 1. As of yet, there are no such case reports of subjects presenting with psychosis in association with such a lipoma.

Lipomas rarely compress or displace the adjacent neural tissue, and they have been hypothesized to encase the nerves and vessels involving the surrounding structures to give rise to a variety of symptoms (17). Symptoms like headache, seizures, loss of consciousness, cranial nerve palsy, behavioral abnormality including aggressive behavior have been attributed to similar quadrigeminal lipomas without any pressure or mass effect in previous case reports (1, 7, 8, 12 and 17). Behavioral changes including aggressive behavior in quadrigeminal cistern lipoma have been hypothesized due to involvement of midbrain- limbic system of Nauta (17, 26). It includes several structures located at the upper dorsal midbrain including mesencephalic reticular system, the periventricular grey matter, and the midbrain-limbic system (17, 27). Moreover, mutism has also been reported in epidermoid cyst in the quadrigeminal cistern region in the literature, suggesting that some anatomical substrate is present in this area that can induce such symptoms (28). Visceral (Tactile) hallucinations have been reported in a patient with tumor of thalamus (29), which is anatomically near the current site of the lesion. In this case, we hypothesized that the appearance of psychotic symptoms is due to similar entrapment of neural tissue by the quadrigeminal plate lipoma.

However, since surgical removal of the lipoma was not planned in this case, due to the absence of any compressive effect and difficulty in operating such tumors due to ensheathment of the surrounding tissues, the exact cause effect relationship of the tumor with the symptoms could not be commented upon with certainty. The fact that the symptoms responded to medical treatment does not prove the absence of any relation between the lipoma and the symptoms, as earlier reports indicated psychotorpics are effective in treating psychotic symptoms and catatonia with identifiable medical or neurological illness (30). Previous reports of headache, loss of consciousness, seizures attributed to similar lipomas have also responded well to medical management (1, 7, 12 and 17). On the contrary, previously reported oculo-motor apraxia and seizures due to similar lipomas remained unchanged even after total or partial removal of the tumor (21, 31). Therefore, the relationship between intracranial lipomas and its symptoms may not be as linear as that of a mass effect relationship. The idea behind reporting this case is to add to the body of the literature a very rare condition with an even more atypical associated presentation.
